# Investigating the effect of electrode orientation on irreversible electroporation with experiment and simulation

**DOI:** 10.1007/s11548-022-02618-y

**Published:** 2022-04-22

**Authors:** Girindra Wardhana, Nivedha M. Raman, Momen Abayazid, Jurgen J. Fütterer

**Affiliations:** 1 Department of Robotics and Mechatronics, University of Twente, Hallenweg 15, Enschede, Overijssel 7500 NH The Netherlands; 2 Department of Medical Imaging, Radboud University Medical Center, Geert Grooteplein Zuid 10, Nijmegen, Gelderland 6525 GA The Netherlands

**Keywords:** Irreversible electroporation, Finite element method, Electrode configuration, Vegetal model

## Abstract

****Purpose**:**

In recent years, irreversible electroporation (IRE) has been developed to specifically destroy undesirable tissues as an alternative to surgical resection. In this treatment, placing multiple electrodes in parallel is required to create a uniform electric field distribution. The process of maintaining parallel electrodes is challenging, and the effect of the electrodes’ orientation accuracy has not been investigated quantitatively. This study investigates the impact of the electrode orientation along with various electrode and pulse parameters on the outcomes of IRE.

****Methods**:**

The electrode configurations that were considered were parallel, forward, and sideward orientation. A numerical model was developed to study the effect of electrode orientation on the electric field distribution, which was validated experimentally on potato tubers as it has similar properties to biological tissue. In addition, a conductivity test was performed to evaluate the conductivity and electroporation threshold of the potatoes.

****Results**:**

The developed numerical model was validated by comparing the electroporated volumes between potatoes from the experiment and simulation, which achieved a mean dice score of $0.727\pm 0.046$. The potato has an electrical conductivity of 0.044–0.454 S/m with an electroporation threshold of 375 V/cm. ANOVA test showed that the difference in the electroporated regions obtained between a parallel orientation and a 5$^{\circ }$ forward and sideward orientation was not significant.

****Conclusion**:**

This study showed that the developed numerical models were validated and able to predict the outcome of IRE on potatoes. In addition, a 5$^{\circ }$ tolerance on the electrode orientation can be defined to obtain a similar response to the parallel orientation.

## Introduction

Irreversible electroporation (IRE) is a novel non-thermal tumor ablation technique which has emerged as a promising ablation technique for solid tumors. IRE is based on exposing the cell to electric pulses, which increases the permeabilization of the cell membrane. The transmembrane potential is then dependent on the strength of the external electric field to which the tissue is exposed to. The electroporation pulse can therefore have either no effect on the cell membrane, reversibly open the cell membrane, nor irreversibly open the cell membrane leading to cell death via apoptosis [1]. The main advantages of this treatment method include reduced damage to the surrounding tissues and vascular complications, ease of application, and reduced risk of heat sink effect [2].

Despite the advantages of IRE, it is not considered as the best choice for tumor treatment. The lack of a systematic way to obtain an optimal treatment planning may cause incomplete tumor ablation and consequently increase the risk of recurrence of the tumor. The effectiveness of the IRE depends on the electric field distribution in the tissue. The electric field distribution is affected by various factors such as the electrode parameters (electrode orientation, active length of the electrode, distance between electrodes, etc.), pulse parameters (number of pulses, pulse width, pulse amplitude, etc.), and tissue properties which influence the dose of the treatment.

One of the standard requirements of IRE for a uniform electric field distribution and maximum ablation volume is that the electrodes must be placed parallel to each other, and the electrode insertion depth must be equal [3]. Maintaining this configuration can be challenging especially when dealing with deep seated tumors.

Several attempts have been made to study the effect of non-parallel electrode placement on the IRE outcomes. However, there has been no detailed investigation to what extent the electrode can be oriented and still be safe for treatment. As a result, the main aim of this study is to investigate the effect of the orientation of electrodes on the electric field distribution. The relation between electrode orientation and other IRE parameters is also investigated in this study, including the electrode active length, distance between the electrodes, pulse number, and pulse length. 3D numerical models were designed and validated to mimic the IRE experiment. The final goal is to find the maximum deviation from parallelism of the electrodes that still gives similar electroporation outcome with respect to the configuration with parallel electrodes. This will support the clinician’s decision for re-inserting the electrodes if the electrodes are not fully parallel.

## Related work

One of the main aspects of treatment planning in IRE is the treatment protocol that is delivered to the patient. Several studies have been reported regarding the influence of treatment protocols on the outcomes of IRE. This section provides a compilation of literature regarding the challenges in treatment planning with regard to probe placement.

The challenge in numerical modeling of clinical electroporation in regard to the ablation of liver tumors was addressed in [4]. The study investigated the model sensitivity to parameters, including the influence of a small translation or inclination of a needle on the electric field distribution. A significant impact on the electric field distribution was observed. Translation of 3 mm produced a Hausdorff distance of 3.8 mm and the difference in volume was 209 $\text {mm}^3$. An inclination angle of 5$^{\circ }$ produced a Hausdorff distance of 4.4 mm and the difference in volume is 247 $\text {mm}^3$. The results show that a small error in the needle location can lead to large errors in the prediction of treatment region.

A unicentric retrospective analysis where the influence of needle is positioning on ablation success of IRE was studied by [5]. For the analysis, fifteen ablations with residual tumor after IRE of hepatocellular carcinoma were identified. Thirty successful ablations after IRE were considered for comparison. Results showed that in patients with residual tumor, the tumor center to ablation center distance and tumor to needle distance were significantly higher, needle depth was too short (2.1 mm vs. 6.8 mm), and the mean needle divergence was significantly higher (7$^{\circ }$ vs. 3.7$^{\circ }$). Because it was a retrospective study, needle parameters could not be varied for further investigation of incomplete ablation of the tumor.

The study regarding the electric field distribution for non-parallel electrodes has been discussed by [6]. The effect of electrode position was evaluated for inclinations at 5$^{\circ }$ and 30$^{\circ }$ in numerical models and experiments. Experiments were done in potato tubers. The numerical study was performed for linear and nonlinear conductivity cases. It was observed that the change in conductivity and electrode orientation affects the electric field distribution, where the maximum of electric field was higher in nonlinear conductivity and non-parallel electrode cases. However, it is still not known whether there is a safety tolerance for needle orientation or not.

Non-parallel placement for needle configuration can also be achieved by having a different insertion depth. The effect of this parameter has been studied by employing a 3D numerical model to optimize the electrode configuration parameters, such as electrode distance, insertion depth, and the number of electrodes [7]. Results showed that V-IRE (the voltage required to electroporate 95% of the tumor) increased as the electrode distance increased. V-IRE values were the lowest for the two electrodes and highest for the three electrodes. It was concluded that electrode distance had a more significant impact on the IRE outcome than the insertion depth.

## Methods

**Fig. 1:**
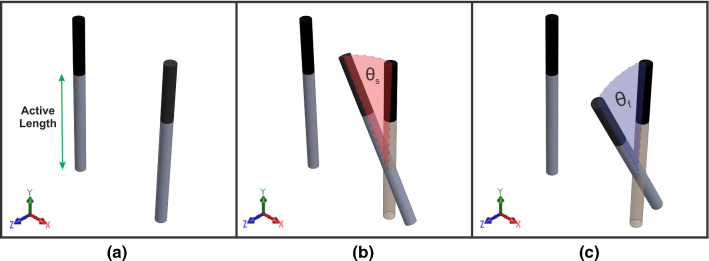
Electrode placement used in the experiments and finite element model, **a** parallel, **b** sideward, and **c** forward orientation

Three types of electrode placements were studied, parallel, sideward, and forward orientation (Fig. 1). For forward and sideward orientation, the electrode was rotated around the center of its active length with a range of 5$^{\circ }$–15$^{\circ }$. Only two electrodes were considered in this study, where the orientation can occur on one or on both electrodes.

Finite element model (FEM) was developed to analyze the effect of electrode orientation on the outcomes of IRE. A set of experiments were conducted to validate this model. The first experiment was about the conductivity measurement of the tissue. Then, electroporation was applied to the tissue with various electrode placements. The electroporated volumes were measured and compared with the results from numerical simulation for model validation using dice score as an evaluation metric. Once the model was validated, the FEM was extended to study the relation between the electrode orientation with various electrode and pulse parameters of IRE. The parameters that were studied for each electrode orientation and its range are summarized in Table 1. The parameter range was defined according to the range used in IRE treatment. Finally, a two-way ANOVA test was performed to understand the interaction between the parameters.

**Table 1:** The electrode and pulse parameter levels used in the parametric study

Parameter	Levels
Electrode distance (mm)	10 mm, 15 mm, 20 mm
Active length (mm)	10 mm, 20 mm, 30 mm
Pulse length ($\upmu$s)	50 $\upmu$s, 70 $\upmu$s, 100 $\upmu$s
Pulse number	50, 70, 90

In this study, IRE experiments were performed in a potato tissue. Potato was chosen due to its distinct properties that can help to assess the IRE outcome. In potatoes, the electroporated region becomes darker 12 h after electroporation [8]. This occurs through oxidation due to the release of polyphenol oxidase that causes membrane rupture. In addition, the potato tissue exhibits a significant increase in conductivity after electroporation, mimicking the response seen in vivo.

### Finite element models

Three-dimensional models were designed using COMSOL Multiphysics v5.5 (Comsol AB, Stockholm, Sweden). This software supports fully dynamic analysis and multi-physics modeling with defined boundary conditions. Previous studies [9–11] have used it as a finite element solver to compute the electric field distribution for simulating the IRE process.

In the 3D model, the geometry shape was simplified to reduce the number of mesh elements for faster computation. The potato tuber was modeled as an ellipsoid, and the two electrodes were modeled as a cylinder with a diameter of 1 mm and inserted into the center of the potato. BTX Gemini X2 (Harvard Apparatus, USA) was used as a pulse generator in this study to produce a series of square wave pulses with a pulse frequency of 1 Hz. The generated pulses were different than the ideal square wave, where the pulse amplitude required some time to rise and fall. Therefore, the electric pulses in the simulation were modeled as square wave pulses with smoothing in the transition zone to mimic the real pulse from the pulse generator. For the conductivity and electric field threshold of the potato, the values were derived from the conductivity test, while for other potato properties, they were obtained from literature. Structural steel was defined as the material of the electrodes, where its properties were predefined in COMSOL. The properties of potato and electrode are summarized in Table 2.

In COMSOL, the electrode orientation and various IRE parameters were configured by adjusting their values within the model. The distribution of the electric field in the potato was computed using the Laplace’s equation:

1 $$\begin{aligned} \nabla \cdot (\sigma (E)\nabla \varphi ) = 0 \end{aligned}$$

where $\sigma$ is the electrical conductivity of the potato as a function of electric field and $\varphi$ is the applied electric potential. To complete the definition of the model, appropriate boundaries were established, including electrically insulating the potato tissue from the external environment.

**Table 2:** Electrical properties of potato and electrode for numerical model

Parameter	Value	References
*Potato*		
Heat capacity ($c_p$)	3780 J/kg K	[12]
Tissue density ($\rho$)	1100 $\text {kg/m}^3$	[13]
Thermal conductivity (*k*)	0.562 W/mK	[12]
Metabolic heat generation ($q^m$)	2161 W/$\text {m}^3$	[14]
*Electrodes*		
Heat capacity $c_p$	840 J/kg K	
Thermal conductivity (*k*)	18 W/mK	
Electrical conductivity ($\sigma$)	1e8 S/m	
Relative permittivity ($\epsilon$)	1	
Density ($\rho$)	6450 kg/$\text {m}^3$	

**Fig. 2:**
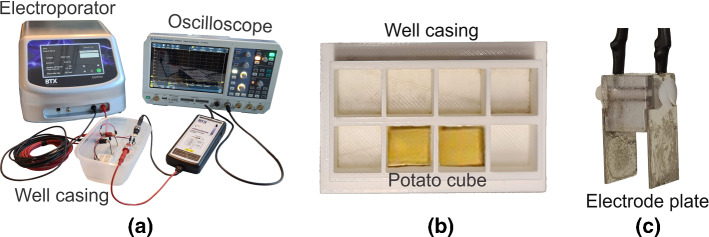
**a** Experiment setup for conductivity test. **b** Well casing printed using 3D printer for placing the potato cube. **c** Parallel plate stainless steel as electrodes for the experiment

### Conductivity test

The electrical conductivity of the potato tissue can be defined as a sigmoid function of the electric field intensity $\sigma (E)$ [15], as indicated by following Eq. 2.

2 $$\begin{aligned} \sigma (E)= \sigma _0 + \frac{(\sigma _1 - \sigma _0)}{2}(1+\tanh {(k_{\text {v}}(E-E_{\text {th}})))} \end{aligned}$$

where $\sigma _0$ and $\sigma _1$ are the conductivity of the non-electroporated and electroporated tissue, respectively. $k_v$ is a fitting coefficient for the sigmoid function. The coefficient is an arbitrary value and used to modify the smoothness of the transition between $\sigma _0$ and $\sigma _1$. $E_{\text {th}}$ is the electric field threshold for electroporation, and E is the applied electric field intensity.

The electric field threshold is the minimum value of the electric field required to electroporate the entire potato cube. These parameter values, $\sigma _0$, $\sigma _1$, $k_v$, and $E_{\text {th}}$, were evaluated by finding the best fit of the pairs $(E, \sigma (E))$ using the nonlinear least squares method with the Curve Fitting Toolbox in MATLAB R2020b (The Mathworks, Inc., Natick, MA, USA) .

Figure 2a shows the setup for the conductivity test. Potatoes were cut into small cubes of 10 mm $\times$ 10 mm $\times$ 10 mm and were placed in a well casing which was printed using a 3D printer. The dimensions of the casing were 11 mm $\times$ 11 mm $\times$ 11 mm (Fig. 2b). Parallel plate stainless steel electrodes were placed on the sides of the potato cube inside the casing. The dimensions of the electrodes were 10 mm $\times$ 20 mm $\times$ 1 mm (thickness). The distance between the plate electrodes was 10 mm (Fig. 2c).

A sequence of 50 square wave electric pulses was applied to the potato cubes via a pulse generator, BTX Gemini X2 (Harvard Apparatus, USA) with a voltage ranging from 100 V to 800 V. To calculate the resistance of the potato cube, a voltage divider circuit was used. A 1 k$\Omega$ resistor was connected across the electrodes and the resistance was calculated from the voltage readings measured by a high-voltage probe which was connected to the digital oscilloscope. Three trials of measurement were performed for each voltage applied. After the resistance value is obtained, the conductivity of potato is calculated using the formula on Eq. 3.

3 $$\begin{aligned} \sigma (E) =\frac{1}{R}\times \frac{L}{A} \end{aligned}$$

where R is the resistance of the potato tuber, L is the width of the potato, and A is the area section of the potato that were connected to the electrode.

To evaluate the effect of the IRE after the experiment, the potatoes were stored in room temperature. The oxidation of the potatoes was visible 24 h after electroporation. The intensity of the discoloration of the potato cube increases with the increase in the electric field. If the electric field value given is beyond the electric field threshold, the potato cubes will turn darker. The visualization of the potato oxidation can help in confirming the obtained threshold from the curve fit.

### Validation experiment

Potato tubers were treated with electroporation using different pulse parameters and electrode orientation. The resulting electroporated volume was used to validate the finite element model. The parameters tested in these experiments are the pulse strength and the number of pulses in a parallel configuration and a variation tilt of 5$^{\circ }$ and 10$^{\circ }$ in the sideward and forward directions. The orientation of the electrodes was adjusted by tilting the electrode from the center of its active length. The electric field distribution is expected to be different for single- and dual-electrode tilt since the electroporation outcome is calculated based on the origin position of the electrode.

A total of thirteen potatoes were used in this experiment, where the pulse parameters and the electrode orientations of each potato are listed in Table 3.

**Table 3:** Pulse and electrode parameter for validation experiment with pulse width 100 µs

Parallel orientation	Sideward orientation	Forward orientation
	(500 V, 50 pulses)	(500 V, 50 pulses)
(1) 500 V, 30 pulses	(6) Single-electrode tilt 5$^{\circ }$	(10) Single-electrode tilt 5$^{\circ }$
(2) 500 V, 50 pulses	(7) Single-electrode tilt 10$^{\circ }$	(11) Single-electrode tilt 10$^{\circ }$
(3) 500 V, 90 pulses	(8) Dual-electrode tilt 5$^{\circ }$	(12) Dual-electrode tilt 5$^{\circ }$
(4) 800 V, 50 pulses	(9) Dual-electrode tilt 10$^{\circ }$	(13) Dual-electrode tilt 10$^{\circ }$
(5) 1000 V, 50 pulses		

**Fig. 3:**
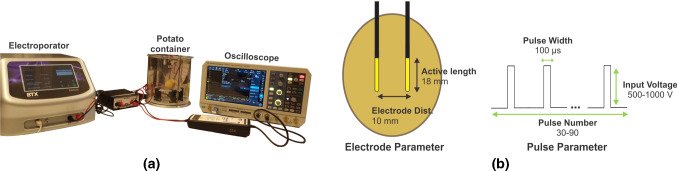
**a** Validation experiment setup used for the electroporation of the potato. **b** Electrode and pulse parameter for the validation experiment

The electroporated volumes (darkened regions) of potatoes were measured by taking MRI images of the potatoes after 24 h of electroporation. The MRI images of the potato were acquired using the MRI scanner in the TechMed center at University of Twente. T2-weighted FLAIR sequence (Table 4) was chosen for the MRI scan based on the study reported [8]. A picture of the experimental setup used for the IRE experiments is shown in Fig. 3a. The active length of the electrodes was 18 mm, and the distance between the electrodes was 10 mm as seen in Fig. 3b.

**Table 4:** MRI scan parameters used for the acquisition of MRI images of electroporated potato tubers (T2-weighted FLAIR sequence)

Parameter	Value
MR acquisition type	3D
Slice thickness	1.1 mm
Inversion time (T1)	1800 ms
Echo time (TE)	168 ms
Repetition time (TR)	7000 ms
Acquisition matrix	200
Echo train length	153
Flip angle	120$^{\circ }$

The electroporation regions obtained from the combination of pulse and electrode parameter in Table 3 were also calculated by using the numerical simulation. The electroporated regions from the experiment and simulation were used for validating the models by measuring the electroporated volume and calculating its dice score. One of the important steps in this dice score calculation is to ensure that the position and orientation of the electroporated region from the experiment and simulation were aligned for an accurate comparison. To achieve this, the electrode position from both results was taken as a reference to facilitate this alignment. The aligned results were then exported to MATLAB for dice score calculation.

## Results

This section describes the experimental results for validating the finite element model and the statistic results from the parametric study.

### Potato characterization

The conductivity of the potato was computed using Eq. 3 and the unknown parameters ($\sigma _0$, $\sigma _1$, $k_v$, and $E_{\text {th}}$) were derived by finding a fit of the experimental data. Each of the conductivity values represents the average of three potato samples.

**Fig. 4:**
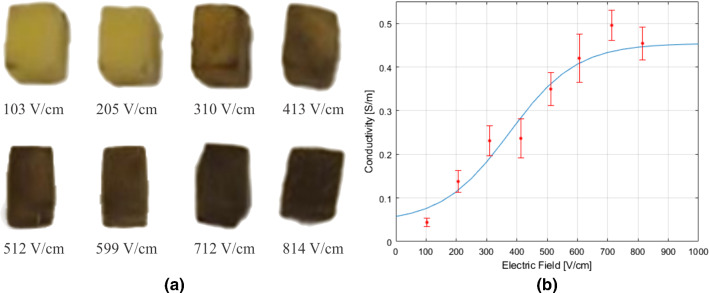
**a** Oxidation of potato cubes seen 24 h after electroporation. **b** Curve fit obtained from the conductivity test. Each point is an average from $n=3$ potato cubes, and the error bar represents the standard deviations

Figure 4a shows the oxidation of the potato cubes treated with various electric field intensities seen 24 h after electroporation. The dark region or the oxidized region represented the electroporated region in the potato and showed an increasing trend with the increase in the electric field intensity. From the curve fit in Fig. 4b, the value for $E_{\text {th}}$ was 375 V/cm and $k_v$ was 0.0045, while the values for $\sigma _0$ and $\sigma _1$ were 0.044 S/m and 0.454 S/m, respectively.

### Model validation

**Fig. 5:**
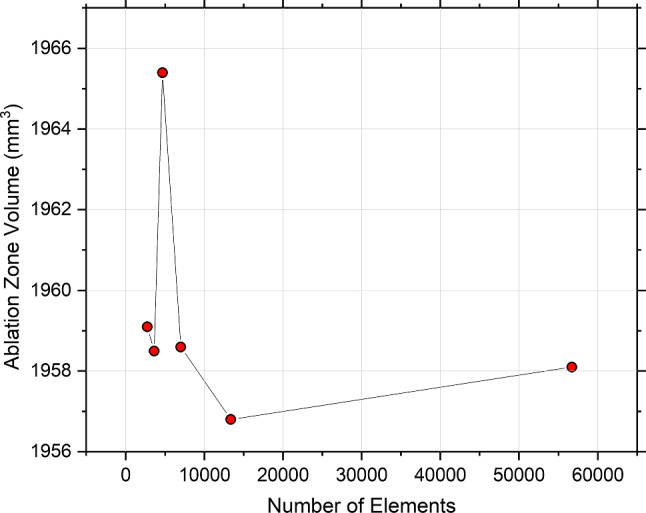
Plot showing ablation zone volume vs. the number of elements for convergence test

For the finite element model, the mesh was built using the physics-controlled mesh option in COMSOL. The mesh resolution was set to normal with a tetrahedral shape element. Convergence test was performed to determine the element size for potato and electrodes. In this test, a sequence of 50 square wave pulses with a voltage of 800 V, pulse width 100 µs, and frequency of 1 Hz was applied to the model. By configuring the parameters in the mesh option, the mesh was refined until the change in volume area of the ablation is within 0.1% difference between two consecutive meshes. From the convergence test result in Fig. 5, the ablation volume change was found less than 0.1% when the number of elements is more than 6987. Therefore, the number of elements in the mesh model was set to 6987. The element sizes (average edge length of elements) for the potato and electrode domains were set to a maximum of 12.0 mm and 0.55 mm, and a minimum of 2.16 mm and 0.37 mm, respectively.

**Fig. 6:**
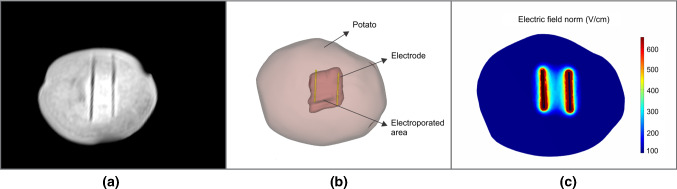
**a** MRI image of one electroporated potato tuber. **b** 3D reconstruction in Slicer for potato, electrodes, and electroporated area from the MRI image. **c** Predicted electric field distribution (V/cm) on the potato from finite element model simulation

Figure 6a shows one of the MRI images of the potato after electroporation. The MRI images were loaded on Slicer v4.11 for obtaining the segmentation of the entire potato, electroporated volumes, and electrodes. In Slicer, MRI images were manually segmented by selecting the pixel threshold for every region. Electrodes were represented by the dark line over the potato region, while the electroporated area has a brighter intensity region around the electrodes. Painting tool was further used to refine these segmentation regions.

Figure 6b presents the 3D reconstruction of the segmentation on one of the potatoes. The reconstructed electrode and potato were then imported to COMSOL to calculate the electroporated volume from the simulation model. The conductivity values $\sigma _0$ and $\sigma _1$, obtained from the conductivity test were used in the simulations. The electric field distributions for the corresponding pulse protocol used in the validation experiments were calculated for each potato. Figure 6c shows the electric field distribution obtained in one of the slices from one of the potatoes.

**Fig. 7:**
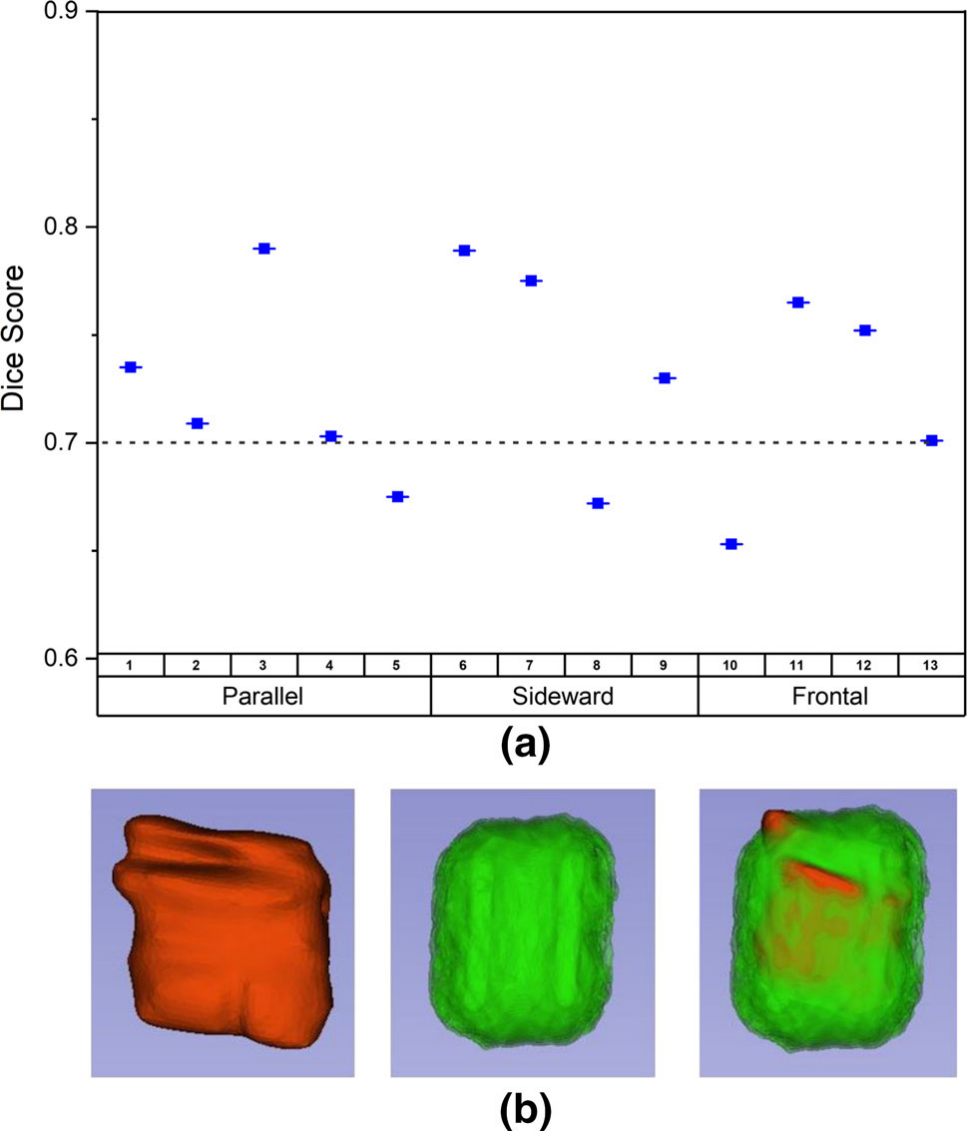
**a** The dice score obtained from thirteen potatoes in validation experiment. **b** Reconstruction of electroporated volume from one of the potatoes with result from MRI (red), simulation (green), and the overlapped between them

The dice score of all thirteen potatoes that were tested in this experiment is shown in Fig. 7. According to the result reported in [16], they found the error in electroporated area calculation for potato with $\Delta$V = 500 V was around 25% and becomes bigger with an increase of the applied voltage. Therefore, it is safe to assume that a dice score of 0.7 can be defined as a threshold for a good match for model validation in this experiment.

By taking the average of all dice scores of thirteen potatoes, the model achieved a good result with a mean dice score of 0.727 ± 0.046. Therefore, it is possible to affirm that the models were validated and can be used in the parametric study.

### Parametric study

The numerical model was further extended to investigate the electric field distribution for different electrode placements (parallel, sideward, and forward orientation) at different parameter levels which are mentioned in Table 1. For each test, two pulse strengths were applied, 1000 V and 3000 V.

**Fig. 8:**
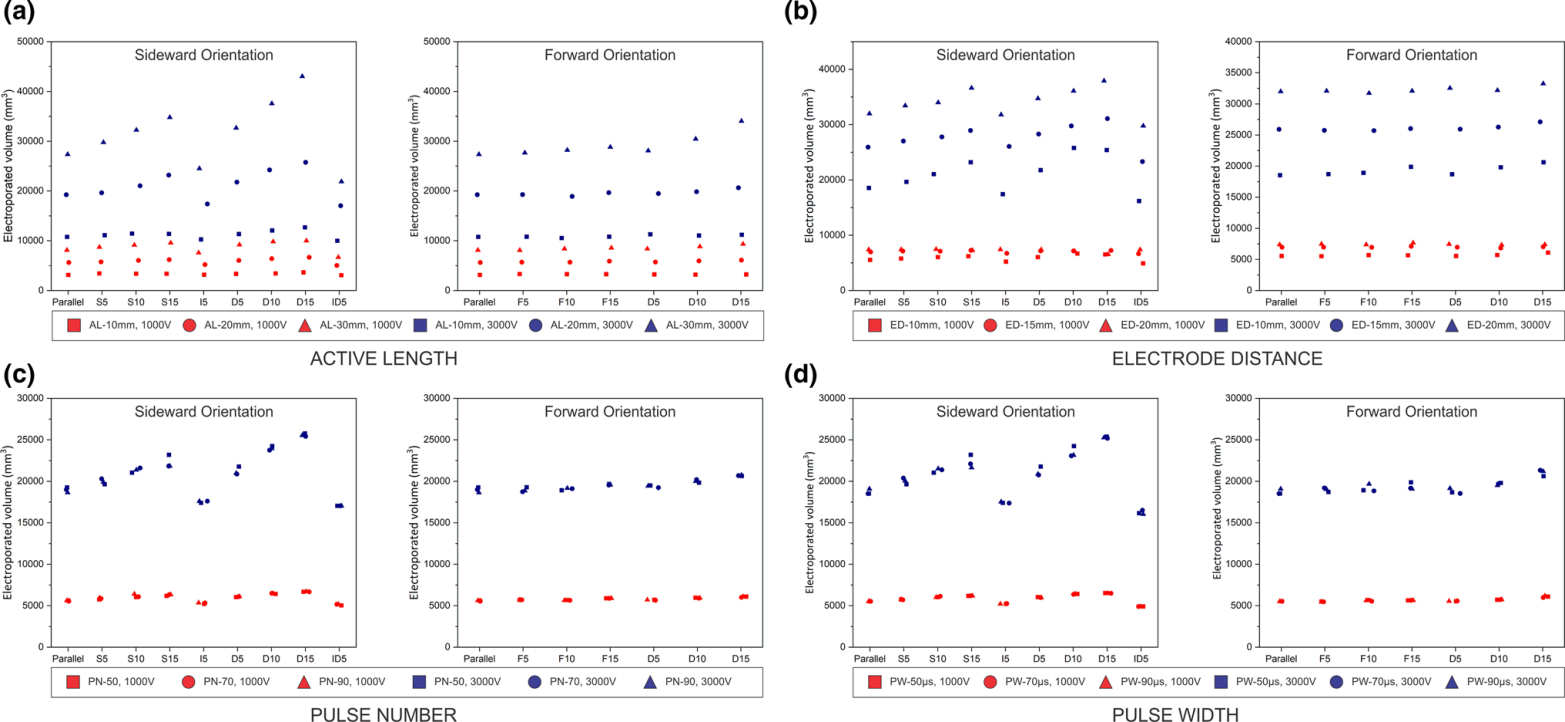
Electroporated volume for sideward and forward orientation with various IRE parameters: **a** active length, **b** electrode distance, **c** pulse number, and **d** pulse width

The electrode parameters included the active length (AL) and the electrode distance (ED). In AL test (Fig. 8a), the electroporated volume increased to 57% for sideward orientation with the dual-electrode setup 15$^{\circ }$ and AL of 30 mm. From ED test (Fig. 8b), maximum increase of 39% in electroporated volume was observed in a dual-electrode setup with 10$^{\circ }$ sideward orientation and ED of 10 mm.

For pulse parameters, pulse number (PN) (Fig. 8c) and pulse width (PW) (Fig. 8d) were tested. The increase in electroporated volume obtained with the variation of PN was less than 1% for both orientations. A similar result was obtained when the PW was varied. Therefore, it can be concluded that the changes in PN and PW were not significant in changing the electroporated volume results.

To further understand the obtained results, an analysis of variance (ANOVA) was performed using IBM SPSS Statistics 27. The results from ANOVA showed that the electrode distance ($p<0.001$), active length ($p<0.001$), and electrode orientation ($p<0.001$) had a significant influence on the electroporated volume. The interaction between the parameters electrode distance and electrode orientation had a significant effect on the electroporated volume only in the single-electrode sideward orientation set up ($p=0.023$), whereas the interaction between active length and electrode orientation had a significant influence on the electroporated volume in all electrode orientations ($p<0.001$).

It was also seen that the difference in the electroporated volume obtained in a parallel orientation and a single electrode with 5$^{\circ }$ orientation in sideward ($p=0.134$), forward ($p=0.276$), and dual electrode with 5$^{\circ }$ orientation in frontal orientation ($p=0.997$) was not significant when the electrode distance was varied. When the active length and electrode orientation were considered, the difference in the electroporated volume obtained between parallel orientation and a 5$^{\circ }$ orientation was not significant in all electrode orientations.

## Discussion

From the parametric study, it can be seen that the parameters such as electrode distance, active length, and electrode orientation had a considerable influence on the increase of electroporated volume. Regarding the electrode orientation, the sideward electrode orientation showed the maximum increase in the electroporated volume.

Statistical results showed that up to 5$^{\circ }$ orientations (sideward and forward direction) can yield a similar result in the electroporated volume as in a parallel orientation. Orienting both electrodes shows a significant increase in the electroporated volume. This electrode arrangement may be considered for deep seated tumors where a parallel orientation of the electrodes is not possible. However, aligning the electrodes toward each other must be avoided due its high risk of over current which may cause thermal damage to the tissue. For tumors where a parallel electrode orientation is possible, a tolerance level of up to 5$^{\circ }$ can be assumed to be safe.

Three-dimensional numerical models were designed to simulate two-needle electrodes placement on potato tissue. Electrode and pulse parameters for IRE were taken from the protocols used in clinical practice to mimic the real treatment scenario. The numerical model was validated by comparing the electroporated regions obtained from experiments performed on potato with the one from simulation. From the thirteen potatoes tested, we successfully obtained a mean dice score of $0.727\pm 0.046$. However, some samples presented a lower dice score in the range of 0.65–0.70.

One of the main reasons for a lower dice score is the difficulty to measure the electroporated region in potatoes. In this paper, the electroporated areas in potato images were manually segmented based on the discoloration in the MRI images. Since the area border was not very clear, there is a possibility that the treated areas were not segmented precisely. Moreover, shrinkage of potato was observed after the treatment, which may lead to a reduction of the segmented electroporated region.

Another reason that may influence the accuracy of the model is the conductivity value and the electric field threshold of the potato. From the conductivity test, the obtained results were similar to the experiments reported in [17]. However, potato has a diverse conductivity as seen from the experiment results of previous studies, such as [16] and [18]. In addition, the electric field thresholds of the potato mentioned in the literature were also varied from 184 V/cm [18] to 478 V/cm [17]. This wide range of conductivity values and electric field thresholds becomes a challenge to design an accurate numerical model for predicting the electroporated volume.

## Conclusions and future work

IRE is a relatively new non-thermal tumor ablation technique. Despite its advantages, it is not considered as an immediate choice of treatment. One of the reasons for this is the lack of a systematic method for optimizing treatment planning. The study of the effect of electrode orientation on the outcomes of IRE is a step forward in the improvement of treatment planning. Maintaining a parallel configuration can be challenging, especially when IRE is used to treat deep seated tumors.

Our study investigated the effect of electrode orientation along with several IRE parameters on the IRE outcome using a 3D numerical model. The study showed that parameters such as active length of the electrodes, electrode distance along with electrode orientation had a significant impact on the electric field distribution. The statistical analysis revealed that the difference in electroporated volumes obtained for a parallel orientation and 5$^{\circ }$ electrode orientation was not significant in most of the orientations. Therefore, it can be inferred that a tolerance of up to 5$^{\circ }$ can be defined to obtain a similar result to that of a parallel orientation in a potato model.

To improve the accuracy of the current model, validation can be performed using hydrogels which mimic the soft tissue more accurately. Hydrogels are a transparent material, so locating and orienting electrodes can be monitored easier, which can improve the results and ensure that the electrodes are inserted in the desired location [9]. In addition, confirmation on different heterogeneous tissues with various IRE configurations is needed to validate the model, since potato tuber is rather homogeneous compared to human or animal tissue. Numerical models can also be further extended to implement more number of electrodes. Finally, few studies have investigated the optimal dose of IRE parameters at a parallel orientation. Optimization of the IRE parameters for different electrode orientations can be useful to better predict the ablation region in treatment planning.
